# Aptamer-functionalized stir bar sorptive extraction for selective isolation, identification, and determination of concanavalin A in food by MALDI-TOF-MS

**DOI:** 10.1007/s00604-023-05795-y

**Published:** 2023-05-13

**Authors:** María Vergara-Barberán, Mónica Catalá-Icardo, Ernesto F. Simó-Alfonso, Fernando Benavente, José Manuel Herrero-Martínez

**Affiliations:** 1 Department of Chemical Engineering and Analytical Chemistry, Institute for Research on Nutrition and Food Safety (INSA•UB), University of Barcelona, C/Martí i Franquès 1-11, 08028 Barcelona, Spain; 2 Department of Analytical Chemistry, University of Valencia, C/Doctor Moliner 50E, 46100 Burjassot, Valencia Spain; 3 Instituto de Investigación para la Gestión Integrada de Zonas Costeras, Campus de Gandia, Universitat Politècnica de València, C/Paranimf 1, 46730 Grau de Gandia, Valencia Spain

**Keywords:** Affinity sorbent, Allergenic protein, Modified PTFE magnet, MS analysis, Sample preparation

## Abstract

An aptamer-functionalized stir bar sorptive extraction (SBSE) coating is described for the first time devoted to selective isolation and preconcentration of an allergenic food protein, concavanalin A (Con A), followed by matrix-assisted laser desorption/ionization mass spectrometry (MALDI-TOF-MS) determination. For this purpose, the polytetrafluoroethylene surface of commercial magnetic stir bars was properly modified and vinylized to immobilize a thiol-modified aptamer against Con A via straightforward “thiol-ene” click chemistry. The aptamer-functionalized stir bar was employed as SBSE sorbent to isolate Con A, and several parameters that can affect the extraction efficiency were investigated. Under the optimized conditions, Con A was extracted and desorbed during 30 and 45 min, respectively, at 25 °C and 600 rpm. The SBSE MALDI-TOF-MS method provided limits of detection of 0.5 μg mL^−1^ for Con A. Furthermore, the SBSE coating was highly selective to Con A compared to other lectins. The developed method was successfully applied to the determination of low levels of Con A in several food matrices (i.e., white beans as well as chickpea, lentils, and wheat flours). Recoveries ranged from 81 to 97% with relative standard deviations below 7%. The aptamer-based stir bars presented suitable physical and chemical long-term stability (1 month) and a reusability of 10 and 5 extraction cycles with standards and food extracts, respectively. The developed aptamer-affinity extraction devices open up the possibility of developing novel highly selective SBSE coatings for the extraction of proteins and peptides from complex samples.

**Graphical abstract:**

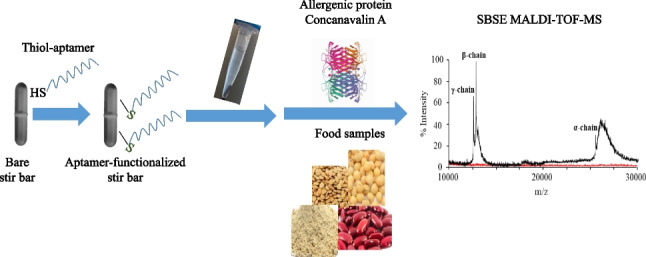

**Supplementary Information:**

The online version contains supplementary material available at 10.1007/s00604-023-05795-y.

## Introduction

Food allergy has become an important and global health problem, being considered as the “second wave” of the allergy epidemic [1]. Within food allergens, dietary lectins pose a potential threat to consumers, and some of them have been included in regulations for labeling allergenic ingredients in foods [2, 3]. However, there is a variety of foods where different allergenic lectins are present, which are not regulated or labeled. For this reason, it is crucial the development of reliable analytical methods to detect and quantify allergenic protein components in foods. These methods need to meet high analytical demands (such as selectivity and sensitivity, among others) and, in most cases, an efficient sample treatment is crucial. In this context, microextraction techniques have opened new possibilities for sample preparation in the last decades due to their well-known advantages [4]. Some of them have been applied for purification of proteins and peptides such as (micro) solid-phase extraction (SPE) [5, 6], liquid-phase microextraction [7], solid-phase microextraction (SPME) [8], and magnetic solid-phase extraction (MSPE) [9, 10], among others. However, some of these sorbents show limited selectivity or small active surface area, which reduce the ability to efficiently extract large biomolecules, such as proteins. Besides, most of these extraction microdevices (such as pipette tips, microcartridges, and fibers) are prone to be clogged or fouled with complex food samples, hindering an appropriate operation.

Stir bar sorptive extraction (SBSE) has been successfully applied to effectively extract low levels of small organic compounds in complex samples [11]. However, it is regretful that there are no reports devoted to the application of SBSE to large biomolecules, such as proteins. This can be due to the potential fouling of the SBSE coating resulting from protein adsorption during extraction and the lack of selectivity of most of the available commercial or home-made coatings, which are typically based on polydimethylsiloxane (PDMS). In the last years, efforts have been paid to demonstrate the applicability in SBSE of molecular biorecognition elements, such as antibodies, molecularly imprinted polymers (MIPs), or aptamers. Among them, antibodies showed high cost of production, as well as stability and cross-reactivity issues. Regarding MIPs, notable challenges still remain, related to preparation against large biomolecules or non-specific binding, among others.

Recently, single-stranded DNA or RNA aptamers have appeared as a new generation of affinity ligands for different bioanalytical purposes [12, 13]. Due to their advantages such as high selectivity and affinity, great reproducibility, superior stability, versatile target binding, and low cost of synthesis and modification, they represent a promising alternative to antibodies or other biorecognition elements. In the last years, several aptamer-based sorbents have been successfully developed for the selective extraction of a wide range of compounds, from small molecules to biomacromolecules, such as proteins, or even biological entities (e.g., cells) [12, 14–17]. However, there are few reports focused on the development of aptamer-functionalized SBSE coatings, being all of them addressed to small organic compounds, specifically persistent organic pollutants [18, 19].

**Fig. 1:**
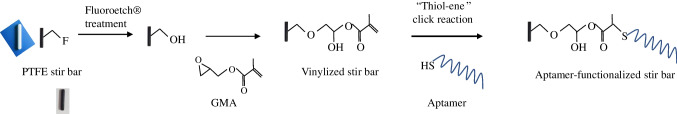
Preparation scheme of the aptamer-functionalized PTFE stir bar

In order to fill the gap of SBSE devices dedicated to proteins, an aptamer-based SBSE coating for isolating and enriching proteins from complex samples is here developed, using the lectin Con A as model allergen protein [20, 21]. To our knowledge, this is the first application of aptamer-functionalized stir bars addressed to the extraction of proteins. For preparation of the aptamer-based SBSE coating, the surface of a commercial polytetrafluoroethylene (PTFE) stir bar was firstly vinylized to assure the subsequent covalent attachment of a thiol-aptamer against the target protein (Fig. 1). Then, the resulting aptamer-based stir bar coating was characterized and evaluated as SBSE sorbent for the extraction of Con A. For detection of the extracted proteins, matrix-assisted laser desorption/ionization mass spectrometry (MALDI-TOF-MS) was selected, due to its potential for accurate identification and determination. The developed SBSE MALDI-TOF-MS method was validated and applied to the isolation, identification, and determination of Con A in food matrices at the typical health-based intake limits established as reference doses for allergenic proteins [22].

## Experimental

The details of reagents and materials, instrumentation, preparation of aptamer-functionalized stir bars, and food sample pretreatment are given in the Electronic Supporting Material (ESM).

### SBSE protocol

The SBSE unit was firstly conditioned with 1 mL of water for 5 min. Water was discarded and a certain sample volume was added. The sample volume was set at 1 mL for Con A standard solutions or 0.5 mL for food protein extracts, and the extraction was performed during 30 min at 600 rpm and 25 °C. After that, the stir bar was washed with 0.5 mL of water for 5 min to remove non-retained compounds, and elution was carried out using 100 or 50 μL (in the case of Con A standard solutions or food protein extracts, respectively) of 100 mM NH_4_OH (pH 11.2) for 45 min at 600 rpm and 25 °C. The eluate was subjected to MALDI-TOF-MS analysis. After each extraction, the stir bar was regenerated by washing with the eluent (100 mM NH_4_OH) and water (0.5 mL for 5 min at 600 rpm and 25 °C, each one). Then, it was stored in water at 4 °C until the next extraction cycle.

### MALDI-TOF-MS

Mass spectra were recorded in mid mass positive mode within a range of 5000–30,000 m/z. Data acquisition and data processing were performed using the 4000 Series Explorer^TM^ and Data Explorer® software (Applied Biosystems), respectively. Sample-MALDI matrix mixtures were freshly prepared as described in a previous work [23]. Briefly, the preparation consisted on depositing onto a stainless steel MALDI plate the following layers: 1 μL of SA in 99:1 (v/v) acetone:water (final SA concentration 27 mg mL^−1^), 1 μL of sample solution, again 1 μL of sample solution (to increase sample homogeneity), and, finally, 1 μL of SA acid in 50:50 (v/v) ACN:water with 0.1% (v/v) of TFA (final SA concentration 10 mg mL^−1^). Spots were allowed to dry at room temperature between the addition of each layer to ensure maximum homogeneity and, therefore, reproducibility in the MALDI-TOF-MS analyses. Three replicates (spots) of each sample were prepared and analyzed.

## Results and discussion

### Preparation and characterization of aptamer-functionalized stir bars

The application of aptamers in SBSE format has been scarcely explored, and specifically for the analysis of small persistent organic pollutants [18, 19]. Apart from the benefit of providing higher selectivity to the extraction process, it would also be interesting to develop inexpensive and robust aptamer-functionalized SBSE coatings from different materials, as an alternative to those that can be prepared in commercial PDMS-coated stir bars (e.g., Twister SBSE Gerstel) [24]. In this context, our research group have successfully employed low-cost commercial PTFE stir bars to prepare SBSE coatings for the extraction of low levels of acidic drugs [4] and estrogens [25] from environmental water and urine samples. Inspired by these studies, PTFE stir bars were selected and properly modified prior to functionalization with the selective aptamer against the target protein Con A. As shown in Fig. 1, the PTFE stir bar was firstly chemically etched with sodium naphthalene (Fluoroetch®) to convert the C-F bonds into C-H, C-OH, and COOH moieties. Then, the incorporated hydroxyl groups were susceptible to react with the epoxide groups of the GMA molecules, giving as a result a vinylized active surface [4, 25, 26], which was functionalized with the thiol-modified aptamer against Con A by a “thiol-ene” click reaction.

**Fig. 2:**
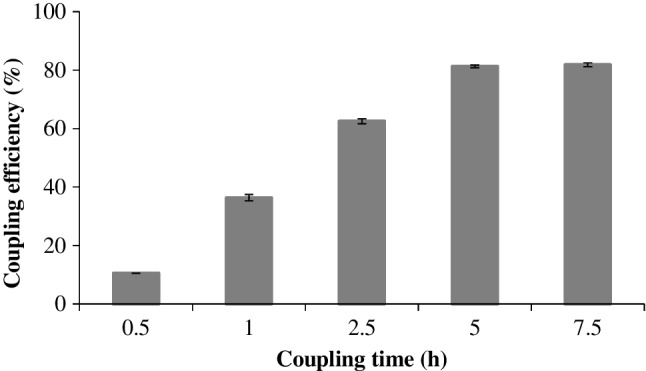
Effect of the thiolated aptamer coupling time on the functionalization of the vinylized PTFE stir bar. Error bar = SD (*n* = 3)

The immobilization of the thiol-modified aptamer onto the vinylized PTFE stir bar was optimized with regard to the coupling time, from 30 min to 7.5 h. The coupling efficiency was evaluated by comparing the aptamer concentration in the coupling solution before and after the immobilization reaction. As shown in Fig. 2, the amount of immobilized aptamer increased during the first 5 h, reaching a plateau after this time, with an average coupling efficiency of approximately 80%.

The preparation of the aptamer-functionalized SBSE coating was also monitored by FT-IR spectroscopy (Fig. S1). As it can be seen, the bare PTFE stir bar (Fig. S1A) gave the characteristic bands of C-F bonds (from 1100 to 1300 cm^−1^). After etching and vinylization treatment, the resulting FT-IR spectrum (Fig. S1B) presented a broad absorption band at 3300 cm^−1^ (corresponding to OH vibrations) and bands at 1600–1700 cm^−1^ attributable to vinyl groups. After immobilization of the thiolated aptamer (Fig. S1C), a slight decrease in the absorption peak corresponding to the C=C was observed at 1600–1700 cm^−1^, jointly with the emergence of a small band at 950 cm^−1^ attributable to C-S vibration. Furthermore, phosphorous was determined by ICP-MS to corroborate the attachment of aptamer onto the stir bars. Thus, the aptamer-functionalized SBSE coating provided significantly greater contents of phosphorus (0.0498 ± 0.0008%), than control vinylized coating (0.0055 ± 0.0003%). The residual phosphorous found in the control coating was probably due to a cross-contamination associated with the reagents and water necessary for the coating preparation.

### MALDI-TOF-MS

**Fig. 3:**
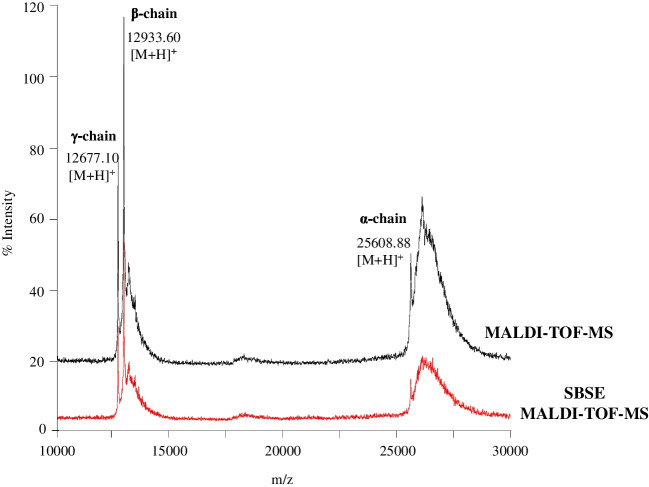
MALDI-TOF mass spectra of a 100 μg mL^−1^ Con A standard solution (black line) and a 5 μg mL^−1^ Con A standard solution after SBSE (red line)

Before the optimization of the SBSE protocol, Con A standard solutions were analyzed by MALDI-TOF-MS. As can be seen in Fig. 3 (black line) for a 100 μg mL^−1^ Con A standard solution, three different proteoforms from Con A were identified, which corresponded to the full length α-chain (M_r_ theorical (M_r_ theo) 25,598.19) and its derived β-chain (M_r_ theo 12,936.36) and γ-chain (M_r_ theo 12,679.85) fragments [27, 28]. The MALDI-TOF-MS method was linear between 10 and 200 μg mL^−1^ of Con A and the protein was detected until 2.0 μg mL^−1^.

To corroborate that the mixtures of proteoforms detected in the Con A standard were the same as in a real food sample, an extract obtained from commercial jack beans (25-fold diluted), which presents high contents of Con A (*ca*. 1.2 mg kg^−1^), was subjected to MALDI-TOF-MS analysis (Supplementary material, Fig. S2).

### SBSE optimization

Several extraction parameters that can affect the extraction performance of Con A were optimized after SBSE MALDI-TOF-MS, including loading solution pH, extraction time, stirring rate, extraction temperature, elution volume, time, and temperature. Extraction efficiency was calculated considering the peak heights of the three proteoforms identified in the mass spectra, as the ratio between the protein concentration in the eluate and in the starting sample. Along the optimization study, an aqueous solution (1 mL) containing 5 μg mL^−1^ of Con A was used as test solution. All experiments were performed in triplicate. The respective text and figures regarding the optimization are included in ESM (Figs. S3 and S4). The following experimental conditions were found to give the best results: (i) loading solution, water; (ii) extraction time, 30 min; (iii) stirring rate, 600 rpm; (iv) extraction temperature, 25 °C; (v) elution volume, 0.05 mL of 100 M NH_4_OH (pH 11.2); (vi) elution time and temperature, 45 min and 25 °C, respectively.

### Selectivity of aptamer-functionalized stir bars

**Fig. 4:**
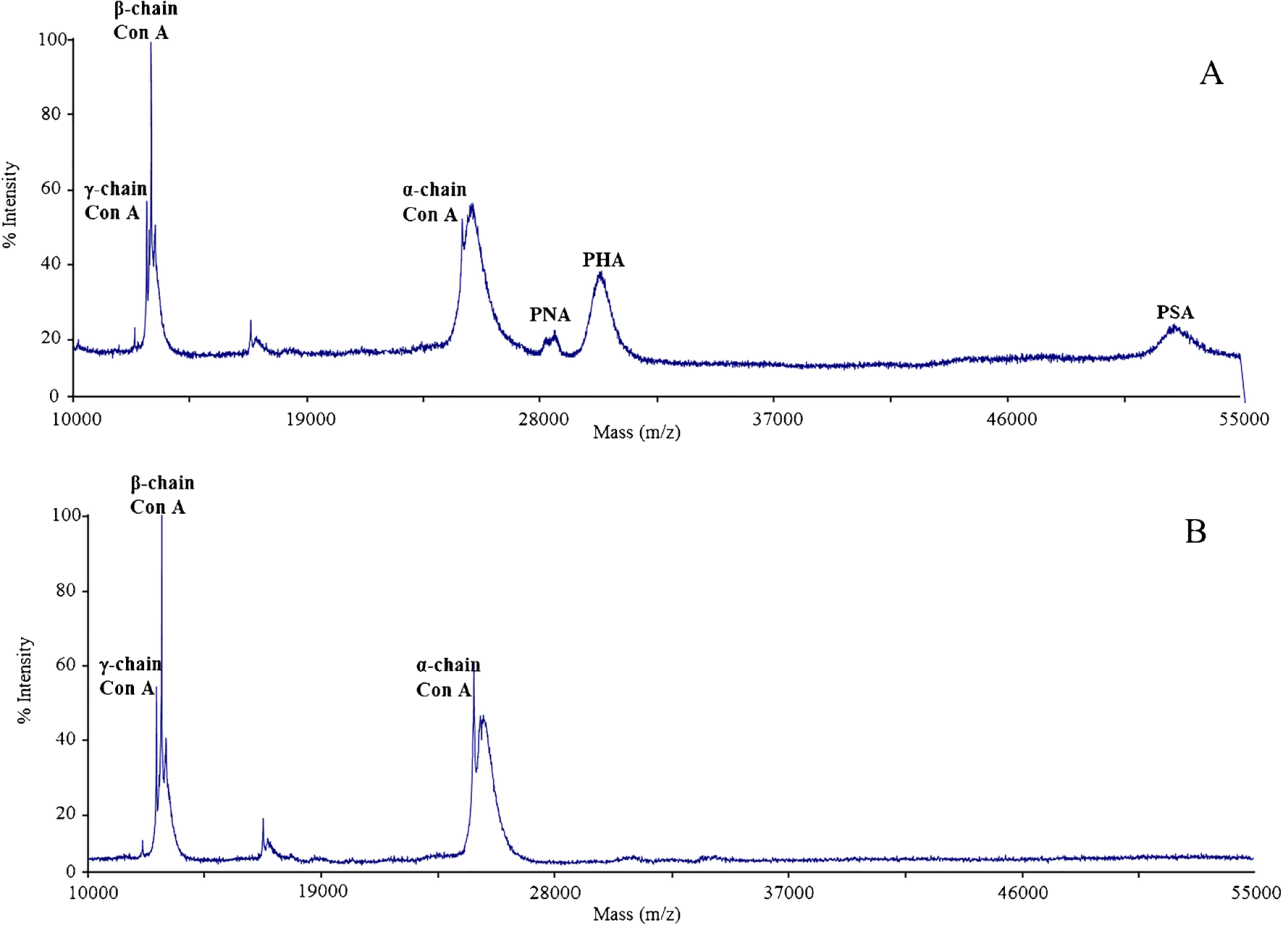
MALDI-TOF mass spectra of a mixture containing PNA, PHA, and PSA lectins at concentration of 10 μg mL^−1^ and Con A at 5 μg mL^−1^ before (**A**) and after (**B**) the aptamer-functionalized SBSE protocol

To evaluate the selectivity of the aptamer-functionalized stir bars, a mixture containing Con A at 5 μg mL^−1^ and three different lectins (i.e., peanut agglutinin (PNA), phytohemagglutinin-L (PHA-L), and *Pisum sativum* agglutinin (PSA)) at 10 μg mL^−1^ was subjected to SBSE MALDI-TOF-MS analysis. As can be observed in Fig. 4A, the MALDI-TOF mass spectrum of the mixture before the extraction showed the presence of Con A proteoforms (α, β, and γ chains), as well as PNA, PHA, and PSA lectins with molecular mass values of around 28,000, 29,500, and 50,000, respectively [29–31] (Fig. 4A). In contrast, after the SBSE pretreatment (Fig. 4B), only the molecular ions corresponding to the Con A proteoforms were observed in the eluate. These results demonstrated the high selectivity of the developed aptamer-functionalized stir bar for Con A even in presence of other lectins.

### Method validation

The developed SBSE MALDI-TOF-MS method was validated in terms of linearity, limits of detection (LOD) and quantification (LOQ), and precision (Table 1). The LOD and LOQ were established at signal-to-noise (S/N) ratios of 3 and 10, respectively. The method was linear for the three Con A proteoforms between 1.5 and 50 μg mL^−1^ of Con A (*R*^2^ > 0.996). The LOD and LOQ values were 0.5 μg L^−1^ and 1.5 μg L^−1^ of Con A, respectively (i.e., they were comprised between 0.16 to 0.18 μg L^−1^ and 0.48 to 0.55 μg L^−1^ for the Con A proteoforms, respectively). The reproducibility of the aptamer-functionalized stir bar preparation procedure was evaluated from the relative standard deviations (% RSD) of the peak heights of the Con A proteoforms for a 5 μg mL^−1^ Con A standard solution. As shown in Table 1, inter-day precision for a single stir bar ranged from 2.7 to 4.5% (*n* = 3), while intra-batch (*n* = 3) and inter-batch (*n* = 3) precision gave %RSD values lower than 5.5 and 7.6%, respectively.

**Table 1:** Figures of merit of the optimized SBSE MALDI-TOF-MS method using the aptamer-functionalized stir bars

	LOD^a^ (μg L^−1^)	LOQ^a^ (μg L^−1^)	Precision (RSD, %)		
			Inter-day^b^	Inter-batch^c^	Intra-batch^d^
Con A α-chain	0.18	0.55	3.3	7.3	4.6
Con A β-chain	0.18	0.53	2.7	7.6	4.1
Con A γ-chain	0.16	0.48	4.5	7.4	5.5

^a^LOD and LOQ values calculated using the signal-to-noise (S/N) ratio of 3 and 10, respectively, considering the relative abundance of each Con A proteoforms (36, 34, and 17% for Con A α, β, and γ chains, respectively, calculated dividing their peak heights by their total sum of peak heights (*n* = 3))

^b^Inter-day values (*n* = 3) using a single stir bar

^c^Inter-batch values (*n* = 3) using stir bars from different preparation batches

^d^Intra-batch values (*n* = 3) using stir bars from the same preparation batch

### Analysis of Con A in food samples

**Fig. 5:**
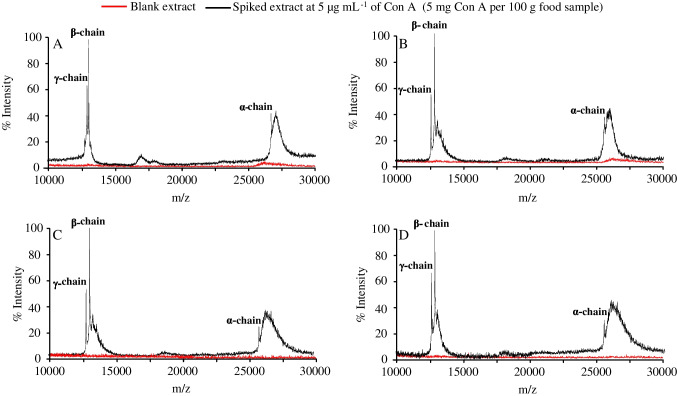
MALDI-TOF mass spectra after aptamer-functionalized SBSE MALDI-TOF-MS analysis of chickpea flour (**A**), lentil flour (**B**), wheat flour (**C**), and white bean (**D**). The red and black lines correspond to blank sample extracts and spiked sample extracts at 5 μg mL^−1^ Con A (5 mg Con A per 100 g of food sample), respectively

The developed SBSE MALDI-TOF-MS method was applied to the analysis of the allergen protein Con A in different food samples, which can be contaminated or contain similar allergenic lectins [32–34]. For this purpose, white beans as well as chickpea, lentil, and wheat flours were studied. A common procedure to isolate lectins from food matrices implies an extraction with 10 mM Na_2_HPO_4_ and 0.5 M NaCl (pH 7.6) [35, 36], which extract not only the target lectins but also other highly abundant proteins. In order to remove interfering proteins, and considering the good thermal stability of Con A, a thermoenrichment pretreatment at 70 °C followed by filtration was applied to both the blank and the spiked protein extracts [16, 17, 36]. After this pretreatment, the three Con A proteoforms were detected by SBSE MALDI-TOF-MS in all the food extracts spiked at 5 μg mL^−1^ of Con A (equivalent to 5 mg Con A per 100 g of food sample), but they were not found in the blanks (Fig. 5). This evidence corroborated the high affinity and selectivity of the aptamer, which was capable to recognize the target analyte even in a wide variety of complex samples.

Moreover, Con A recoveries found in the spiked samples were satisfactory for all food matrices, with values comprised between 81 and 97% (Table 2).

**Table 2:** Con A proteoforms recoveries in spiked food samples (5 mg Con A per 100 g of food sample) after aptamer-functionalized SBSE MALDI-TOF-MS analysis. SD (*n* = 3)

Sample	Con A proteoform	Recovery (%) ± SD
Chickpea flour	α-chain	86 ± 6
	β-chain	97 ± 6
	γ-chain	84 ± 5
Lentil flour	α-chain	81 ± 5
	β-chain	95 ± 4
	γ-chain	93 ± 6
Wheat flour	α-chain	91 ± 7
	β-chain	88 ± 4
	γ-chain	91 ± 5
White bean	α-chain	97 ± 6
	β-chain	90 ± 7
	γ-chain	99 ± 4

Additionally, the lifetime of the aptamer-functionalized stir bars was investigated at 5 μg L^−1^ using Con A standard solutions and spiked food extracts. The results (Fig. S5) showed that the stir bars can be reused (with recoveries higher than 80%) at least 10 and 5 times for standards and food extracts, respectively.

Also, a storage stability study was done with the aptamer-functionalized stir bars after 2 months of storage in water at 4 °C. The results showed no significant changes on performance over this period using 5 μg L^−1^ Con A standard solutions (recoveries > 85%).

All these results demonstrated the great performance of the proposed aptamer-functionalized stir bars. Other benefit is that several PTFE stir bars can be modified simultaneously (*ca*. 10 in 12 h) giving an estimated cost of 0.6 €/stir bar, and up to 16 €/stir bar after the aptamer functionalization, which made the device production cost-effective and potentially feasible to be commercialized, as described for Con A or adapted with other aptamers against other target proteins. In addition, the combination of aptamer-functionalized SBSE with MALDI-TOF-MS presented a good selectivity, low detection limits, and allows a rapid, selective, sensitive, and accurate identification and determination of the target protein, preventing false positives or erroneous quantifications of non-MS-based biosensors or bioassays.

### Comparison with other microextraction methods for the determination of Con A

The proposed aptamer-functionalized SBSE MALDI-TOF method was compared with other microextraction methods reported in literature for the extraction and determination of Con A [37–42]. In general, the developed method showed better pretreatment times than other works reported in the literature [37–41], except to that described by Qu et al. [42]. Concerning performance features such as recovery or LOD, it should be mentioned that the focus of the most studies mentioned in Table 3 was mainly oriented to lectin purification using affinity sorbents combined with spectrophotometric or chromatographic techniques. Indeed, almost in the totality of these works, information regarding these analytical parameters is not mentioned. In fact, the only work that provides recoveries was that developed by Ahirwar et al. [39], being these values significantly lower than those found in our method. Additionally, in most of these studies, the selectivity of the affinity sorbent was not investigated. With regard to the reusability of purification/extraction device, the aptamer-modified coating stir bar also gave similar values to those obtained in other methods. All these results highlighted that the proposed method showed excellent analytical performance as well as a satisfactory applicability to complex samples.

**Table 3:** An overview on recently reported microextraction methods for the determination of Con A

Method	Material	Sample matrix	Extraction time (h)	Recovery (%)/	LODs (μg L^−1^)	Selectivity	Reusability (analyses)	Ref
SPE UV	Cryogel columns modified with GlcNAc	Standard solution	> 15	-	-	No	5	[37]
SPE UV	MIP cryogel columns	Jack beans	> 2	-	-	Yes	10	[38]
SPE UV	Aptamer-functionalized agarose resin column	Jack beans	> 2	66	-	No	7	[39]
DSPE colorimetric assay	GMNM	Jack beans	2.25	-	-	No	-	[40]
DSPE colorimetric assay	MISPs	Jack beans	2.25	-	-	Yes	10	[31]
SPME UV	Silica fibers modified with poly (NIPAAm-*co*-BMA-*co*-MBA) hydrogel	Standard solution	0.17	-	-	No	-	[42]
SBSE MALDI TOF-MS	Aptamer-functionalized stir bar coating	Food samples	1.25	81–97	0.5 μg L^−1^	Yes	5–10	This study

*GMNM*, glucosylated magnetic nano matrix; *MISPs*, surface-imprinted silica particles; *poly (NIPAAm-co-BMA-co-MBA)*, poly(N-isopropylacrylamide-co-butyl methacrylate-co-N,N-methylenebisacrylamide); *DSPE*, dispersive solid-phase extraction

## Conclusions

A novel aptamer-functionalized SBSE coating to selectively isolate and preconcentrate the allergenic food protein Con A followed by rapid MALDI-TOF-MS analysis was developed. Commercial PTFE stir bars were vinylized to attach a thiolated aptamer against Con A via straightforward “thiol-ene” click chemistry. The unique properties of aptamer-functionalized stir bars make them able to selectively recognize the target protein even in presence of other similar lectins from foods (bean, peanuts, and peas). The developed stir bars exhibited several other advantages such as cost-effective and reproducible preparation, long-term stability, and reusability. Although the method requires lengthy incubation/elution times (more than 1 h), a high throughput can be achieved by serial extraction using a thermomixer of multiple vessels. Finally, the combination with MALDI-TOF-MS analysis allowed an improvement of the selectivity and sensitivity of the whole assay, enabling the identification and determination of Con A in complex food samples, at the low levels of the typical health-based intake limits established as reference doses for allergenic proteins. Indeed, the proposed method was successfully applied to the analysis of Con A in white beans as well as chickpea, lentils, and wheat flours with satisfactory recoveries. It is noteworthy that this is the first aptamer-functionalized stir bar proposed for the recognition of large biomolecules and the obtained LOD values are adequate to determine the targeted allergen in foods. The developed method offers a new insight in agri-food, clinical, and environmental fields since it can be extended to other large biomolecules and, in general, to any type of compounds of interest, as long as the corresponding selective aptamers are available.

## Supplementary information

ESM 1: Figures S1–S5 (DOCX 175 kb)
