# Supplementary material for: Aptamer-functionalized stir bar sorptive extraction for selective isolation, identification, and determination of concanavalin A in food by MALDI-TOF-MS
Source: Mikrochim Acta. 2023 May 13;190(6):219. doi: 10.1007/s00604-023-05795-y (PMC10182934; doi:10.1007/s00604-023-05795-y)
Supplement: Supplementary file 1 — Figures S1–S5 (DOCX 175 kb) [file 604_2023_5795_MOESM1_ESM.docx]

**Electronic Supplementary Material**

**Aptamer-functionalized stir bar sorptive extraction for selective isolation, identification and determination of concanavalin A in food by MALDI-TOF-MS**

**María Vergara-Barberán^1,2^, Mónica Catalá-Icardo^3^, Ernesto F. Simó-Alfonso^2^, Fernando Benavente^1^, José Manuel Herrero-Martínez^2*^**

*^1^Department of Chemical Engineering and Analytical Chemistry, Institute for Research on Nutrition and Food Safety (INSA•UB), University of Barcelona, C/ Martí i Franquès 1-11, 08028 Barcelona, Spain*

*^2^Department of Analytical Chemistry, University of Valencia, C/ Doctor Moliner 50E, 46100 Burjassot, Valencia, Spain*

*^3^ Instituto de Investigación para la Gestión Integrada de Zonas Costeras, Campus de Gandia, Universitat Politècnica de València, C/ Paranimf 1, 46730 Grau de Gandia, Valencia, Spain*

*Corresponding author: Dr. José Manuel Herrero-Martínez e-mail: jmherrer@uv.es

**Table of contents**

Page S1-S4. Experimental section

Page S5-S10. Results and discussion

Page S11-S12. References

**Reagents and materials**

All the reagents and materials used in this work were of analytical grade unless otherwise stated. PTFE-coated stir bars (6 mm length × 2 mm diameter) were provided by VWR International Eurolab (Barcelona, Spain). Acetic acid (HAc) (glacial), ammonium hydroxide (NH_4_OH, 25%), bovine serum albumin (BSA), sodium dihydrogenphosphate (Na_2_HPO_4_, ≥99.0%), sodium chloride (NaCl, ≥99.5%), Phaseolus vulgaris (PHA-L; from red kidney bean), trifluoroacetic acid (TFA, 99.0 %), acetone (99.8 %), and sinapinic acid (SA, ≥99.0 %) were supplied by Merck (Darmstadt, Germany). Acetonitrile (ACN, LC-MS), ethanol (EtOH, 96%), and methanol (MeOH, HPLC-grade) were purchased from Panreac AppliChem (Barcelona, Spain). Azobisisobutyronitrile (AIBN), glycidyl methacrylate (GMA), phytohemagglutinin-L (PHA-L), peanut agglutinin (PNA), P*isum sativum* agglutinin (PSA) and tris(2-carboxyethyl)phosphine hydrochloride (TCEP) were from Sigma-Aldrich (Steinheim, Germany). N,N-dimethylformamide (DMF), triethylamine (TEA), and water (LC-MS grade) were obtained from VWR Chemicals (Fontenay Sous Bois, France), Alfa-Aesar (Karlsrube, Germany), and Fisher Scientific (Loughborough, UK), respectively. Sodium naphthalene solution (FluoroEtch^®^) for the treatment of PTFE stir bar surface was provided by Acton Technologies (County Limerick, Ireland).

The thiol-modified single-stranded DNA-aptamer against Con A with a C6 spacer arm (5′-SH-(CH_2_)_6_-CGAGTAACGCTGTCTCTTCCGAATCGGGGGAAGGCGGAGGG-3’, 41-mer, molecular mass (M_r_)= 13100) was synthesized and purified by HPLC by Integrated DNA Technologies (Leuven, Belgium). The aptamer was dissolved in nuclease-free water (VWR) to obtain a final concentration of 100 µmol L^-1^.

Con A standard protein purified from jack beans (*Canavalia ensiformis*) and jack beans were provided by Sigma-Aldrich. A 1 mg·mL^-1^ stock solution of Con A standard was prepared in water, then it was aliquoted and stored in the freezer. Aliquots were defrosted before use. Working standard solutions were prepared by dilution in water and stored in the fridge when not in use.

White beans and flours from chickpea, lentil, and wheat were purchased in a Barcelona local market.

**Instrumentation**

A TS-100 thermoshaker (Biosan, Riga, Latvian Republic) was used for sample incubation. Attenuated total reflection Fourier-transform infrared (FT-IR) spectra of the stir bar surfaces were directly recorded on a Tensor 27 FT-IR spectrometer (Bruker, Bremen, Germany) with a DuraSamplIR II accessory from Smiths Detection Inc. (Warrington, UK) equipped with nine reflection diamond/ZnSe DuraDisk plates. The aptamer concentration in the supernatant solution before and after the immobilizing reaction was measured with a DeNovix DS-11 NanoDrop spectrophotometer (Thermo Scientific, Wilmington, EUA). The determination of phosphorous content in bare and aptamer-modified stir bars was done, after removing the Teflon coating using a cutting blade, with a 7900 inductively coupled plasma mass spectrometer (Agilent Technologies, Waldbronn, Germany). Centrifugal filtration was carried out in a cooled Rotanta 460 centrifuge (Hettich Zentrifugen, Tuttlingen, Germany). MALDI-TOF-MS analyses were performed in a 4800 MALDI TOF/TOF mass spectrometer (Applied Biosystems, Waltham, MA, USA).

**Preparation of aptamer-functionalized stir bars**

The chemical modification of the PTFE stir bar was adapted from a previous study [1]. First, the stir bar was immersed in the commercial etchant solution (Fluoroetch^®^) and stirred at 60 ºC in a water bath for 30 min and 200 rpm under nitrogen stream to produce hydroxyl groups onto the PTFE surface (**Fig. 1**). After that, the stir bar was sequentially washed with MeOH, water, and 1% (v/v) HAc solution at 65 °C in a water bath at 200 rpm and oven-dried at 60 ºC for 2 h. Then, the vinylization reaction of the hydroxyl groups of the stir bar surface was carried out under the following reaction conditions: 2 M GMA in DMF containing 5 mM TEA (pH 8.0), at 60 °C in a water bath for 2 h and 200 rpm. Vinylized stir bar was then rinsed with acetone and oven-dried at 60 ºC for 1 h. Several vinylized stir bars, typically 10, can be simultaneously prepared using volumes of reagents, solvents, and solutions of 20 mL in a 100 mL two-neck flat bottom flask. This volume ensured a complete stir bar immersion and a proper agitation. Before aptamer immobilization onto the surface of vinylized stir bars, 50 µL of the aptamer solution (100 µmol L^-1^) was pretreated with TCEP to reduce the disulfide bond and release the thiol group, according to the manufacturer’s aptamer reduction protocol [2]. Then, aptamer folding treatment was done by heating the solution at 95 ºC for 10 min and cooling at 4 ºC for 10 min in a thermoshaker. The synthesis of the aptamer-functionalized SBSE coating was carried out by “thiol-ene” click reaction according to a previous work [3], but with modifications. Each vinylized stir bar was immersed in 1 mL solution containing 0.9 % (m/v) AIBN (prepared in ACN:H_2_O (30:70 (v/v))) and 50 µL of 100 µmol L^-1^ solution of the activated thiolated aptamer were added. The mixture was stirred gently at 400 rpm for 5 h and 55 ºC. The aptamer concentration in the supernatant solution before and after the immobilizing reaction was estimated by measuring the absorbance at 260 nm. Finally, the aptamer-functionalized stir bar was washed with MeOH and water and air-dried. The SBSE unit was used immediately or was stored in water at 4°C until use.

**Food sample pretreatment**

Jack and white beans samples were milled in a mixer grinder to obtain a fine powder. Two grams of ground jack or white beans or flour from chickpea, lentil, or wheat were mixed with 20 mL of 10 mM Na_2_HPO_4_ containing 0.5 M NaCl at pH 7.6 [4,5]. The mixture was kept under magnetic stirring overnight at room temperature. After that, it was centrifuged at 10,000×*g* for 10 min at 4 ºC, and an aliquot of the resulting supernatant (2 mL) was incubated at 70 ºC for 2 h to remove non-thermostable proteins [5]. Then, the suspension was centrifuged at 10,000×*g* for 10 min at 4 ºC and the supernatant was filtered through a 0.22 μm polyvinylidene difluoride centrifugal filter (Ultrafree-MC, Millipore, Bedford, MA, USA). Afterwards, 500 µL of this filtrate was spiked with an appropriate volume of 100 µg mL^−1^ Con A standard solution to reach a final concentration of 5 mg Con A per 100 g of food (excepting jack bean extracts, which were not spiked but just 25-fold diluted). Finally, low molecular mass (M_r_) compounds were removed with 3,000 M_r_ cut-off cellulose acetate centrifugal filters (Amicon Ultra-0.5, Millipore) as described in previous studies [6,7]. Briefly, the sample solution was centrifuged at 10,000×g for 10 min at 25°C, and the residue was washed with 50 µL of water three times under the same conditions. The final residue was recovered inverting in a new vial the upper reservoir and spinning at a reduced centrifugal force (300×g for 2 min). Then, water was added to adjust the final volume to 500 µL before SBSE MALDI-TOF-MS analysis.

**Results and discussion**

**
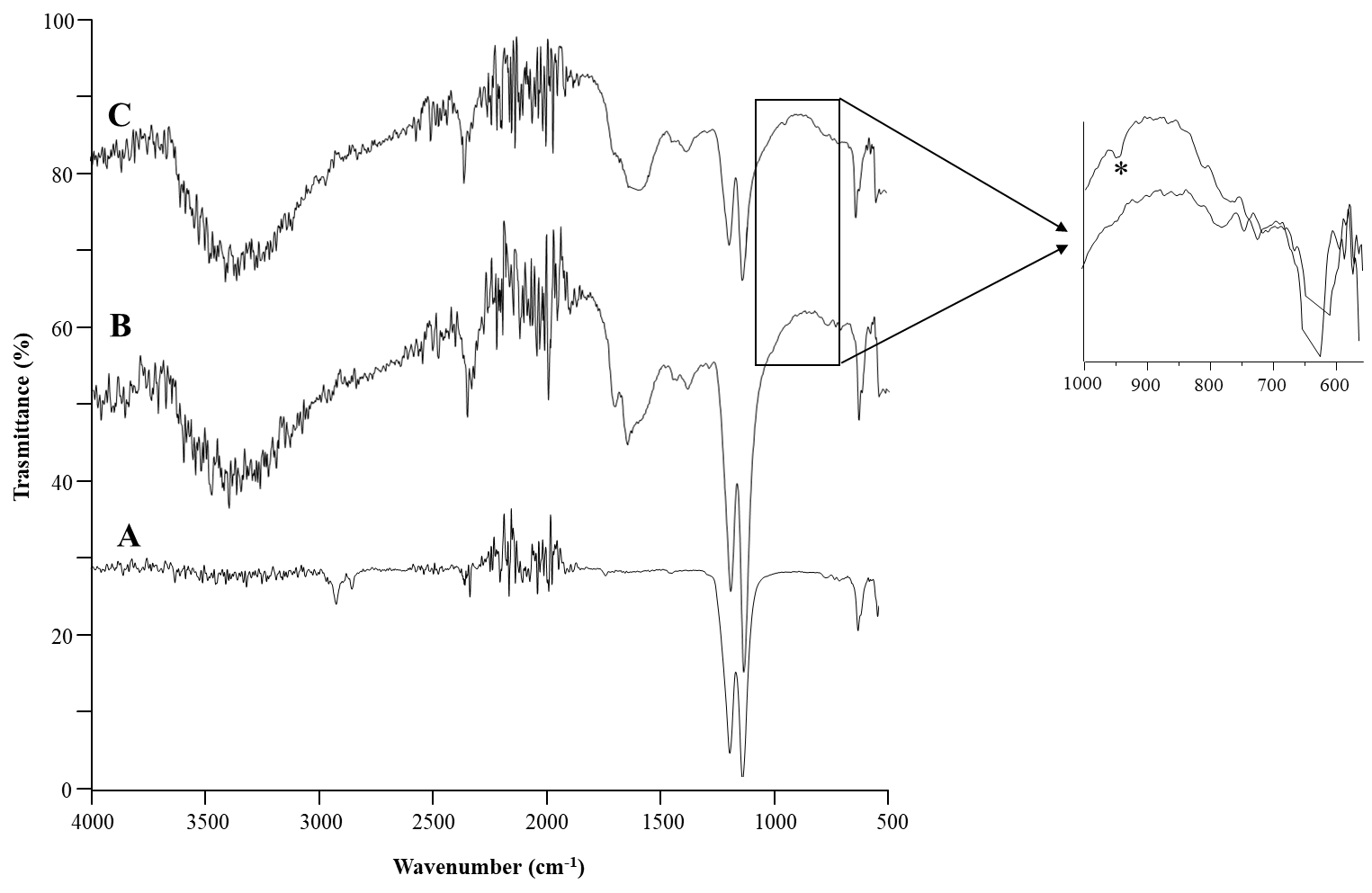
**

**Fig. S1.** FT-IR spectra of bare PTFE stir bar (A), PTFE stir bar after etching with Fluoroetch^®^ and vinilyzation (B), and final aptamer-functionalized stir bar (C).

**
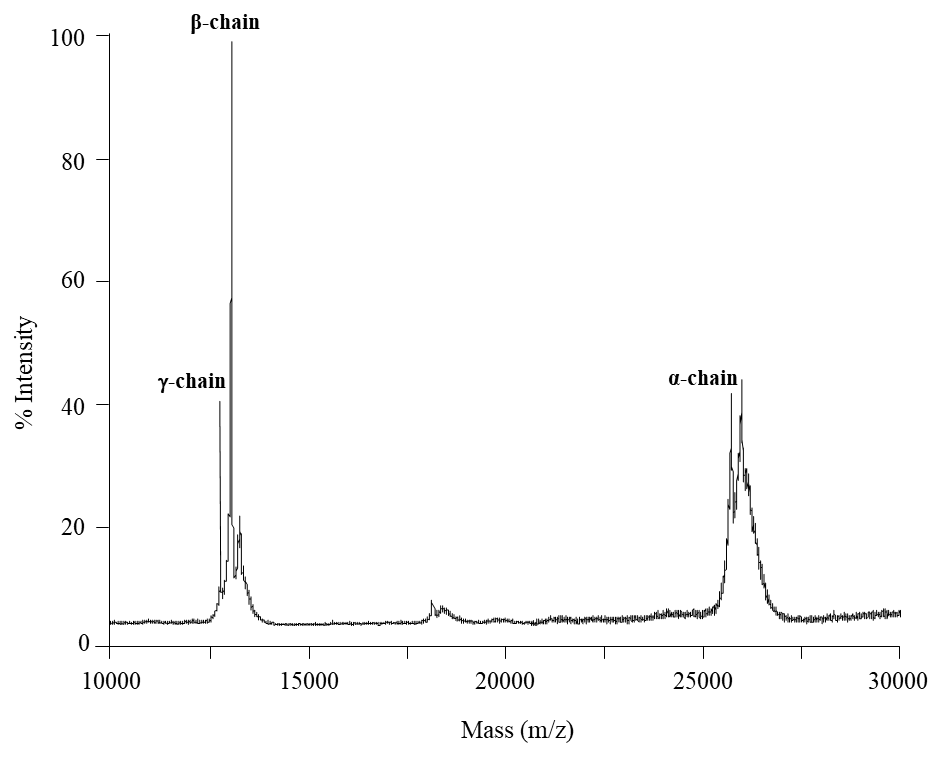
**

**Fig. S2.** MALDI-TOF mass spectra of a 25-fold diluted extract prepared from commercial jack beans.

**SBSE optimization**

First, the effect of the loading solution pH on the Con A retention was evaluated by preparing Con A standard solutions (5 µg mL^-1^) in water, 1 M HAc (pH 2.3), and 10 mM phosphate buffer with 0.5 M NaCl (pH 7.6). In a previous study by on-line solid-phase extraction capillary electrophoresis-mass spectrometry [8,9], water and acidic media provided satisfactory retention of the protein biomarker α-synuclein using a sorbent based on aptamer-functionalized magnetic beads, allowing the detection of low levels of this protein in blood. Also, phosphate buffer solution was considered since it is the typical extractant used to isolate Con A from food samples [4,5]. In view of these considerations, the starting extraction SBSE conditions were as follows: 1 mL of 5 µg mL^−1^ Con A standard solution dissolved in the three abovementioned loading solutions, stirred for 10 min at 600 rpm and 25 ºC. As observed in **Fig. S3A**, the best results in terms of protein retention, analyzing by MALDI-TOF-MS the remaining Con A in the loaded solution, were achieved using water and phosphate buffer (neutral pH conditions) with values in the range of 69.5 to 80.0 % for the three Con A proteoforms. This fact can be explained taking into account that at neutral pH, the aptamer presented its maximum activity and formed a stable ligand-binding domain, which is consistent with the neutral pH of the SELEX process [4,10,11]. For simplicity and the slightly better protein retention, water was selected for subsequent experiments.

The next parameter investigated was the effect of the extraction time. Different extraction times ranging from 10 to 60 min were assayed. As observed in **Fig. S3B**, the maximum retention for all Con A proteoforms was achieved at 30 min, remaining the values almost unchanged after this time. Consequently, 30 min was selected as extraction time for further experiments.

Stirring rate of the extraction was also an important parameter affecting the extraction performance, since low stirring rates could provide poor retention values, whereas high stirring rates could accelerate molecular mass transfer rate and reduce the time to reach the thermodynamic equilibrium. Therefore, different stirring rates were investigated (from 300 to 1000 rpm) (**Fig. S3C**). The results indicated that no obvious variation of the retention of Con A proteoforms was observed in the tested stirring rate range. Considering that a vigorous stirring rate may cause splashing or damage in the aptamer coating, an average stirring rate of 600 rpm was selected for the rest of the study.

The effect of the extraction temperature on the extraction efficiency was also studied from 25 to 55 ºC (**Fig. S3D**), but no significant differences on proteoform retention were observed. Therefore, the extraction temperature was set to 25 ºC for simplicity.

**Fig. S3.** Influence of different factors on the retention of the three Con A proteoforms using the aptamer-functionalized stir bars: loading solution pH (A), extraction time (B), extraction stirring rate (C), and extraction temperature (D). Error bar = SD (n=3).

Next, the elution step of Con A was considered. In this sense, several strategies have been reported to desorb proteins from oligosorbents such as pH variation, increase of temperature and ionic strength, or use of chaotropic agents, among others [12]. In our previous studies with aptamer-functionalized sorbents [8,9], we found that a basic eluent of 100 mM NH_4_OH (pH 11.2) allowed the successful elution of alpha-synuclein and subsequent separation and detection by CE-MS. Indeed, this eluent is also compatible with the volatility requirements for analysis by MALDI-TOF-MS. Using this desorption solution, other elution parameters such as elution volume, time, and temperature were investigated analyzing by MALDI-TOF-MS Con A in the eluted solutions. The desorption volume affected both desorption efficiencies and the enrichment factor (EF) of the SBSE process, being the EF estimated as the ratio between the volumes of starting sample and eluate (e.g. 1 mL/ 0.1 mL = 10). Considering both aspects, elution volumes ranging between 0.05 to 0.5 mL were tested under the following conditions: 0. 5 mL of starting sample, stirring for 30 min at 600 rpm and 25 ºC. Volumes lower than 0.05 mL were not tested since they did not completely cover the stir bar. As observed in **Fig. S4A**, the recovery of the three Con A proteoforms increased slightly from 0.05 mL (30-40%) to 0.1-0.5 mL (40-56%). Therefore, an elution volume of 0.05 mL was chosen as the best compromise between EF and extraction efficiency.

After that, in order to increase the elution efficiency, the elution time and temperature were evaluated. In the first case, elution times from 30 to 70 min were tested (**Fig. S4B**). The results showed that Con A was almost quantitatively recovered when 45 min were selected. Under these conditions, elution temperatures ranging from 25 to 45 ºC were evaluated (**Fig. S4C**). As observed, Con A recoveries slightly decrease with temperature, hence, 25 ºC were also maintained during the elution. **Fig. 3** (red line) shows the MALDI-TOF-MS mass spectra for a 5 µg·mL^-1^ Con A standard solution under the optimized conditions.

**Fig. S4**. Influence of different elution parameters on the recovery of the three Con A proteoforms using the aptamer-functionalized stir bars: elution volume (A), elution time (B), and elution temperature (C). Error bar = SD (n=3).

**
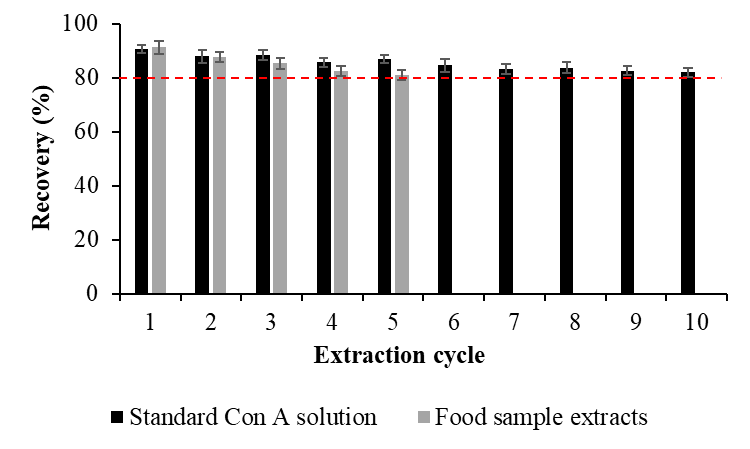
**

**Fig. S5.** Recovery values of a 5 μg L^-1^ Con A standard solution using the aptamer-functionalized stir bar as SBSE sorbent. Values calculated for the α-chain proteoform. Error bar = SD (n=3).

**References**

1. Nadal JC, Catalá-Icardo M, Borrull F, Herrero-Martínez JM, Marcé RM, Fontanals N (2022) Weak anion-exchange mixed-mode materials to selectively extract acidic compounds by stir bar sorptive extraction from environmental waters. J. Chromatogr. A 1663:462748. https://doi.org/10.1016/j.chroma.2021.462748
2. Reduction protocol for thiol-modified oligonucleotides. https://eu.idtdna.com/ pages/support/guides-and-protocols. Accessed 10 January 2023
3. Wang Z, Zhao JC, Lian HZ, Chen HY (2015) Aptamer-based organic-silica hybrid affinity monolith prepared via “thiol-ene” click reaction for extraction of thrombin. Talanta 138:52-58. https://doi.org/10.1016/j.talanta.2015.02.009
4. Ahirwar R, Nahar P (2015) Screening and identification of a DNA aptamer to concanavalin A and its application in food analysis. J Agric Food Chem 63:4104-4111. https://doi.org/10.1021/acs.jafc.5b00784
5. Jiang B, Wang B, Wang L, Lv X, Li D, Liu C, Feng Z (2019) Two-step isolation, purification, and characterization of lectin from Zihua snap bean (*Phaseolus vulgaris*) seeds. Polymers 11:785. https://doi.org/10.3390/polym11050785
6. Pero-Gascon R, Pont L, Benavente F, Barbosa J, Sanz-Nebot V (2016) Analysis of serum transthyretin by on-line immunoaffinity solid-phase extraction capillary electrophoresis mass spectrometry using magnetic beads. Electrophoresis 37:1220-1231. https://doi.org/10.1002/elps.201500495
7. Pont L, Benavente F, Barbosa J, Sanz-Nebot V (2017) On-line immunoaffinity solid-phase extraction capillary electrophoresis mass spectrometry using Fab´antibody fragments for the analysis of serum transthyretin. Talanta 170:224-232. https://doi.org/10.1016/j.talanta.2017.03.104
8. Pero-Gascon R, Benavente F, Minic Z, Berezovski MV, Sanz-Nebot V (2020) On-line aptamer affinity solid-phase extraction capillary electrophoresis-mass spectrometry for the analysis of blood α-synuclein. Anal Chem 92:1525-1533. https://doi.org/10.1021/acs.analchem.9b04802
9. Salim H, Pero-Gascon R, Giménez E, Benavente F (2022) On-line coupling of aptamer affinity solid-phase extraction and immobilized enzyme microreactor capillary electrophoresis-mass spectrometry for the sensitive targeted bottom-up analysis of protein biomarkers. Anal Chem 94:6948-6956. https://doi.org/10.1021/ acs.analchem.1c03800
10. Zeng J, Wang Q, Gao J, Wang W, Shen H, Cao Y, Gan N (2020) Magnetic stir bars with hyperbranched aptamer as coating for selective, effective headspace extraction of trace polychlorinated biphenyls in soils. J Chromatogr A 1614:460715. https://doi.org/10.1016/j.chroma.2019.460715
11. Chu Q, Liu Y, Jiang S, Zhu Y, Lyu H, Xie Z (2021) A novel adsorbent based on aptamer prepared via “thiol-ene” click for specific recognition of phthalic acid esters. Anal Chim Acta 1146:109-117. https://doi.org/10.1016/j.aca.2020.12.039
12. Pichon V (2020) Aptamer-Based and Immunosorbents. In Solid-Phase Extraction. Handbooks in Separation Science; Poole CF, Ed.; Elsevier pp:151-183. https://doi.org/10.1016/B978-0-12-816906-3.00006-6
